# Efficient quantitative monitoring of translational initiation by RelE cleavage

**DOI:** 10.1093/nar/gkac614

**Published:** 2022-07-25

**Authors:** Caroline M Focht, Scott A Strobel

**Affiliations:** Department of Molecular Biophysics and Biochemistry, Yale University, New Haven, CT 06510, USA; Institute of Biomolecular Design and Discovery, West Haven, CT 06516, USA; Department of Molecular Biophysics and Biochemistry, Yale University, New Haven, CT 06510, USA; Institute of Biomolecular Design and Discovery, West Haven, CT 06516, USA; Department of Chemistry, Yale University, New Haven, CT 06511, USA

## Abstract

The sequences of the 5′ untranslated regions (5′-UTRs) of mRNA alter gene expression across domains of life. Transcriptional modulators can be easily assayed through transcription termination, but translational regulators often require indirect, laborious methods. We have leveraged RelE’s ribosome-dependent endonuclease activity to develop a quantitative assay to monitor translation initiation of *cis*-regulatory mRNAs. RelE cleavage accurately reports ligand-dependent changes in ribosome association for two translational riboswitches and provides quantitative information about each switch's sensitivity and range of response. RelE accurately reads out sequence-driven changes in riboswitch specificity and function and is quantitatively dependent upon ligand concentration. RelE cleavage similarly captures differences in translation initiation between yeast 5′-UTR isoforms. RelE cleavage can thus reveal a plethora of information about translation initiation in different domains of life.

## INTRODUCTION

Organisms across all domains of life control gene expression at the transcriptional and translational levels. The sequences of the 5′ untranslated regions (5′-UTRs) have been shown to alter gene expression in prokaryotic as well as eukaryotic systems (1–3). These regulatory mechanisms are prime targets for synthetic biology applications and drug development efforts (4,5), so the connection between RNA motifs and functional outputs must be better understood.

The well-established transcription termination assay can be used to interrogate how transcription of a bacterial gene is impacted by its 5′-UTR *in vitro* (6). Using radiolabeled nucleotide triphosphates, the length differences between prematurely truncated and full-length transcripts can be easily resolved via polyacrylamide gel electrophoresis (PAGE). Translational modulators, however, lack such an established assay. The ideal ribosome association assay would take an instantaneous snapshot of ribosome loading. The sensitive, specific assay should be easily scalable to address varied sequences and initiation conditions.

A variety of different approaches have been used to monitor ribosomal association *in vitro* including toeprinting, ultracentrifugation, filter binding, and *in vivo* fluorescent reporters. Toeprinting uses the extension of radiolabeled primers in a reverse transcription reaction to identify the position of a ribosome on a transcript (7). The bulk of the ribosome acts as a roadblock, halting the progress of the reverse transcriptase and producing a shorter DNA species. The ribosome is then mapped onto the transcript by comparing the shorter DNA products to the full-length sequence on a sequencing gel. Ultracentrifugation, on the other hand, leverages the size of the ribosome to separate bound from free mRNAs in a sucrose gradient (8). The laborious and time-intensive method is not practical for small numbers of sequences and varied assay conditions, as each requires a separate sucrose gradient centrifugation. Filter binding assays separate ribosome-bound mRNAs from free mRNAs via ribosome association with a nitrocellulose membrane (9). Since ribosome association rather than initiation is being measured, however, this approach suffers from non-specific interactions and high background signal. Fluorescent reporters (10) provide valuable *in vivo* data but have limited access to variable condition space when examining responses to compounds with weak solubility or unknown transport mechanisms and metabolism.

Here, we demonstrate that RelE can be utilized to quantify sequence-driven differences in translation initiation. RelE is a ribosome-dependent endonuclease found in bacteria that is used to globally repress translation under stress conditions (11,12). RelE is part of the type II toxin-antitoxin family. It binds and rapidly cleaves mRNAs in the A-site of initiated and elongating ribosomes with a rate constant of 380 s^–1^ (13). In the absence of ribosomes, RelE has no endonucleolytic activity, making it a sensitive and specific measure of mRNA-ribosome association. RelE cleaves the message between the second and third nucleotides and thus defines the mRNA position to nucleotide resolution relative to the ribosomal A-site.

RelE has been previously applied to ribosome profiling studies in bacteria (14). The precision of RelE cleavage was used to locate the A-site of elongating ribosomes and refine the position of ribosome footprints. Thus, RelE cleavage was able to reveal important reading frame information among bacterial transcripts. Here we demonstrate that RelE’s efficiency and precision can be used to analyze specific mRNA regulatory motifs *in vitro*.

We have leveraged RelE’s ribosome-dependent endonuclease activity to develop a quantitative assay for translationally regulated mRNAs. We have validated this method on two translational riboswitches in bacteria with purified components and a commercially available translation system. We have also demonstrated it is applicable to eukaryotic systems by showing it can measure differential ribosome loading based upon yeast 5′-UTR isoforms in a commercially available wheat germ extract. We demonstrate that RelE cleavage is sensitive to subtle sequence changes in both the bacterial and eukaryotic contexts. This RelE cleavage assay can be applied to complex sequence libraries and used to rapidly generate extensive information about the intricacies and functional requirements of regulatory RNAs at the translational level.

## MATERIALS AND METHODS

### Design of RNA constructs

The wild-type sequence for the *Pseudomonas aeruginosa* Guanidine-II riboswitch has been characterized previously (15). The *Pae* WT construct comprised −72 to +27 relative to the start of translation (Supplemental Table S1). The second codon was mutated to a TAG stop codon for efficient RelE cleavage. The wild-type sequence for the *Vibrio vulnificus* adenine-sensing *add* riboswitch was previously reported (16) and adapted for our study. The *Vvu* WT construct comprised −106 to +24 relative to the start of translation (Supplemental Table S1), and the second codon was similarly mutated to a TAG stop codon. The T7 promoter was added to the 5′ end of both sequences for *in vitro* transcription. Full sequences and mutant sequences are provided in Supplementary Materials.

The sequences of the yeast 5′-UTR isoforms were previously reported (8). Each isoform was followed by ∼20 nucleotides of the endogenous coding sequence (Supplemental Table S1). The second codon was mutated to a TAG stop codon.

### RNA preparation and labeling

RNA was transcribed directly off oligonucleotides (Supplemental Table S2) ordered either from Keck Oligo Synthesis Resource at Yale University or Integrated DNA Technologies (IDT) and purified via denaturing 10% PAGE. For 5′ radiolabeling, riboswitch RNA was dephosphorylated with Antarctic Phosphatase (NEB) and labeled with ^32^P-γ-ATP (Perkin Elmer) via T4 PNK (NEB). For yeast 5′-UTR isoforms, RNA was capped with ^32^P-α-GTP via the Vaccinia Capping System (NEB). Radiolabeled RNA was similarly purified via denaturing PAGE or by passing through a G-25 spin column (Cytiva).

### Translation initiation and RelE cleavage

For riboswitch constructs, RNA refolding solutions contained the following: 100 nM RNA, 1X 219H Buffer (50 mM HEPES–KOH pH 7.5, 70 mM NH_4_Cl, 30 mM KCl, 7 mM MgCl_2_), variable concentrations of ligand, and 5′radiolabeled RNA in trace. Guanidine riboswitch RNA was refolded by heating to 90°C for 2 min, slowly cooled to 37°C at a ramp rate of 0.1°C/s, and then held at room temperature. Adenine riboswitch RNA was refolded by heating to 95°C for 5 min and then immediately cooling to 4°C. Translation initiation solutions contained the following: 1X 219H Buffer, 100 nM IF1, 100 nM IF2, 100 nM IF3, 1 mM GTP, 100 nM fMet-tRNA^fMet^, 100 nM 70S *Escherichia coli* ribosomes, and 10 nM RNA. IF1, IF2, and IF3 were purified from *E. coli* as previously described (17). tRNA^fMet^ (MP Biomedicals) was charged with *E. coli* S-100 extract as previously described (18). For the guanidine riboswitch, ligand concentrations ranged between 0 and 25 mM guanidine hydrochloride and 50 mM urea. For the adenine riboswitch, ligand concentrations ranged between 0 and 500 μM adenine or 0 and 5 μM guanine. Translation initiation solutions were incubated at 37°C for either 15 min (guanidine riboswitch) or 30 min (adenine riboswitch) before being incubated with 1 μM RelE. Cleavage reactions were quenched with the addition of an equal volume of formamide loading buffer (FLB).

For reactions in a commercially available protein expression system, 10 nM refolded *Pae* Gdm-II RNA was incubated in the PURExpress ΔRF123 (NEB) system for 30 min at 37°C before being incubated with 1 μM RelE. 5′ radiolabeled RNA was present in trace. Guanidine hydrochloride concentrations ranged between 0 and 25 mM. Cleavage reactions were immediately quenched with the addition of an equal volume of formamide loading buffer (FLB).

For yeast 5′-UTR constructs, trace amounts of radiolabeled RNA were incubated with 50% (v/v) wheat germ extract (Promega), 0.8U/μl RNaseOUT (Invitrogen), 120 mM potassium acetate, 80 μM methionine, and 5 μM RelE for 30 minutes at 25°C. Reactions were then quenched with the addition of an equal volume of FLB.

### Denaturing PAGE and analysis

Radiolabeled products were separated on a 10% polyacrylamide gel, visualized with a Typhoon (GE), and analyzed in ImageQuant (GE). Assuming the concentration of labeled RNA is much less than the *K*_1/2_, the guanidine data were fit in PRISM with the following equation:

(1) $$\begin{equation*}Y\ = \ {\rm amplitude}\ \left[ {\frac{{{X}^n}}{{\left( {{K}^n + \ {X}^n} \right)}}} \right] + Y_{\rm min}\end{equation*}$$

*Y* is the percent cleaved; *X* is the concentration of ligand; *K* is the concentration of half-maximal response; *Y*_min_ is the percent cleaved in the absence of ligand; amplitude is the difference between the *Y*_max_ and *Y*_min_; *n* is the Hill coefficient.

The adenine data were fit in PRISM with the following equation:

(2) $$\begin{equation*}Y\ = \ {\rm amplitude}\ \left[ {\frac{X}{{K + X}}} \right] + \ {Y}_{{\rm min}}\end{equation*}$$

*Y* is the percent cleaved; *X* is the concentration of ligand; *K* is the concentration of half-maximal response; *Y*_min_ is the percent cleaved in the absence of ligand; amplitude is the difference between the *Y*_max_ and *Y*_min_.

For the yeast 5′-UTR constructs, constructs were pairwise compared in PRISM using an unpaired two-tailed *t* test with Welch's correction.

## RESULTS

### Designing the RelE cleavage assay for translational riboswitches

**Figure 1.**
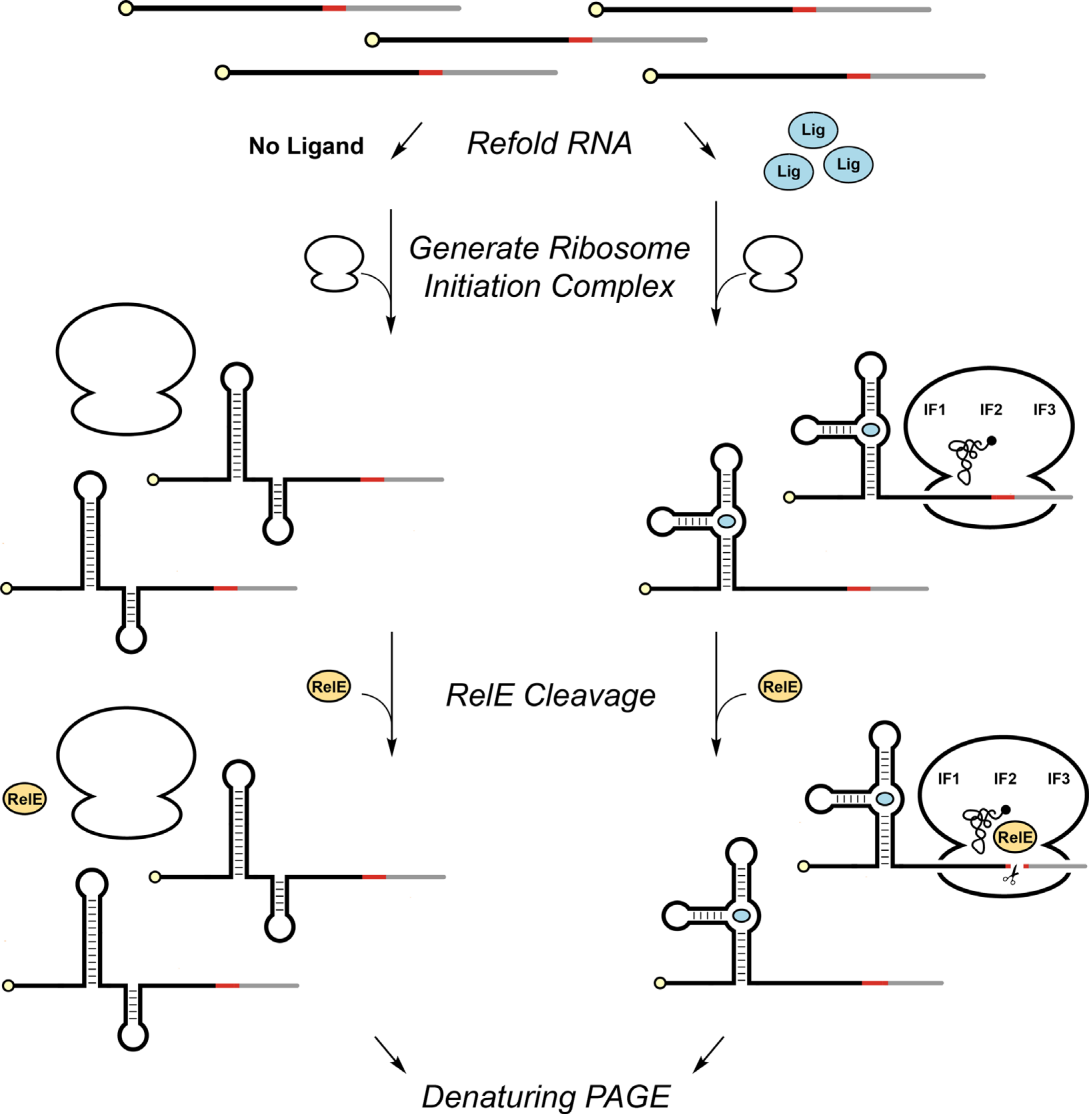
Schematic of RelE cleavage assay for translational riboswitches. Labeled RNA was refolded in the presence or absence of ligand. 5′ ^32^P label is shown in yellow, site of RelE cleavage in red, and downstream sequence in grey. Refolded RNA was used to form ribosome initiation complexes. RelE was used to cleave ribosome-bound RNAs, and the full-length and cleaved species were separated via denaturing PAGE. The radiolabeled full-length and cleaved RNAs were then quantified across ligand concentrations to determine dose-dependent changes in translation initiation.

We developed the following method for assaying translational riboswitch function (Figure 1). *In vitro* transcribed and radiolabeled RNA was refolded with varying concentrations of ligand prior to incubation with the ribosomes and initiation factors (IFs). Initiation complexes that form can neither elongate nor release since only the fMet-tRNA^fMet^ is provided. Following initiation, RelE cleaved the ribosome-bound riboswitch RNA, resulting in a size difference that is ligand-dependent which can be revealed by denaturing PAGE. Percent cleavage of the message was quantified as a function of ligand concentrations to determine dose-dependent changes in translation initiation.

### RelE cleavage reads out ligand-dependent riboswitch ribosome association

**Figure 2.**
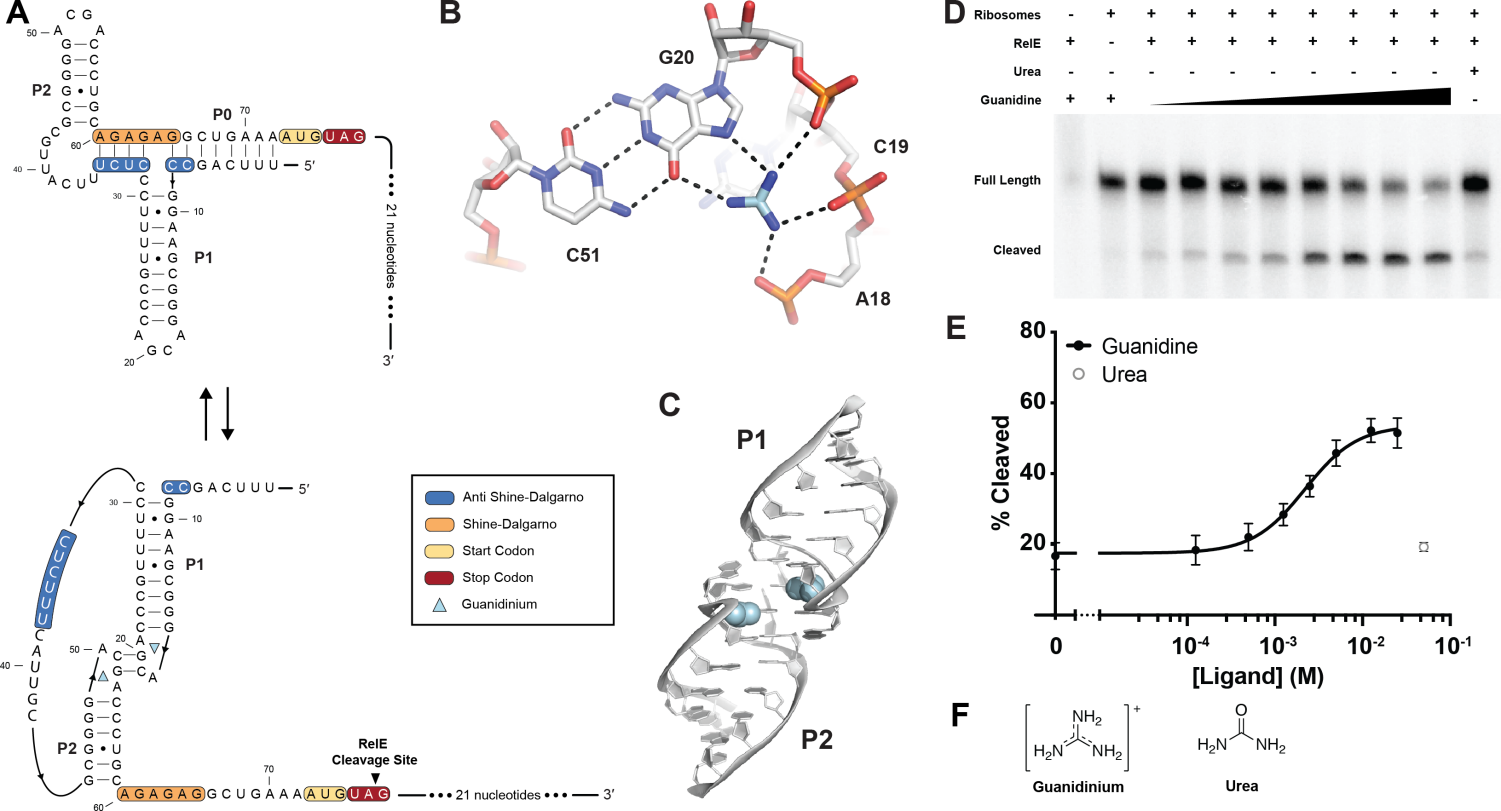
RelE cleavage quantifies ligand-dependent changes in translation initiation. (**A**) The secondary structure of the *Pae* Guanidine-II riboswitch in the ON and OFF states. The anti Shine-Dalgarno is shown in blue, the Shine-Dalgarno is shown in orange, the start codon is shown in yellow, and the stop codon for RelE cleavage is shown in red. (**B**) The P1 binding pocket of the *Pae* Guanidine-II riboswitch. G20 from P1 forms a Watson Crick pair with C51 from P2. The ligand forms hydrogen bonds with the phosphate backbones of A18, C19 and G20 in addition to hydrogen bonds with the Hoogsteen face of G20. (**C**) The crystal structure of the dimerized helices of the *Pae* Guanidine-II riboswitch. The guanidine ligand is shown in light blue (PDB: 5VJ9). (**D**) Gel readout of the *Pae* Guanidine-II RelE cleavage. Guanidine concentrations ranged from 0 to 25 mM. (**E**) The ligand response curve of the *Pae* Guanidine-II riboswitch. F) Chemical structures of guanidinium and urea.

We first tested the sensitivity of RelE cleavage to ligand-dependent conformational changes in the *Pseudomonas aeruginosa* (*Pae*) Guanidine-II riboswitch. This small motif controls the expression of a multidrug resistance transporter (19) and it is one of over 800 examples of this exclusively translational riboswitch class. The riboswitch features two almost identical hairpins, P1 and P2, and binds two molecules of the positively-charged guanidinium ion through a kissing loop interaction (15,20) (Figure 2A–C). The linker between the two hairpins is poorly conserved. Previous modeling of the *P. aeruginosa* Guanidine-II riboswitch (15) suggests that the 5′ and 3′ flanking sequences may hybridize to form a P0 helix (Figure 2A). This P0 helix is proposed to sequester the Shine-Dalgarno sequence and thus moderates the ribosome's access to the RNA, though there has been no direct biochemical evidence of this mode of regulatory control.

We performed the RelE cleavage assay on the wild-type *Pae* Guanidine-II riboswitch over a range of experimental conditions. Ribosome initiation was IF-dependent (Supplemental Figure S1), and cleavage mapped to the A-site. Cleavage was not detectable in the absence of ribosomes, confirming the ribosome dependence of RelE’s endonuclease activity (Figure 2D). Similarly, cleavage did not occur in the absence of RelE. Cleavage was dependent upon the addition of the guanidine ligand and the extent of RelE cleavage was dependent upon the ligand concentration (Figure 2D). The percent cleaved at each concentration was fit with Equation (1) since we expect this system to be cooperative (19), which produced measures of the sensitivity (K_1/2_), dynamic range between OFF and ON states (amplitude), and cooperativity (Hill coefficient) of the system (Figure 2E). The resulting curve indicates that the *P. aeruginosa* Guanidine-II riboswitch responds to guanidine with an apparent *K*_1/2_ of 2.2 ± 0.2 mM, an amplitude of 36 ± 4%, and a Hill Coefficient of 1.5 ± 0.2 (*n* = 4). This sensitivity is within the concentration range expected for this metabolite given the reported affinities of other guanidine riboswitch classes and is consistent with the proposed cooperativity of the system (19,21–24). Cleavage is specific for guanidine binding as no modulation occurs in the presence of urea despite their chemical similarity (Figure 2F). A similar guanidine response profile was observed when translation initiation and RelE cleavage were performed on the wild-type *Pae* Guanidine-II riboswitch in a commercially available protein expression system (Supplemental Figure S2).

**Figure 3.**
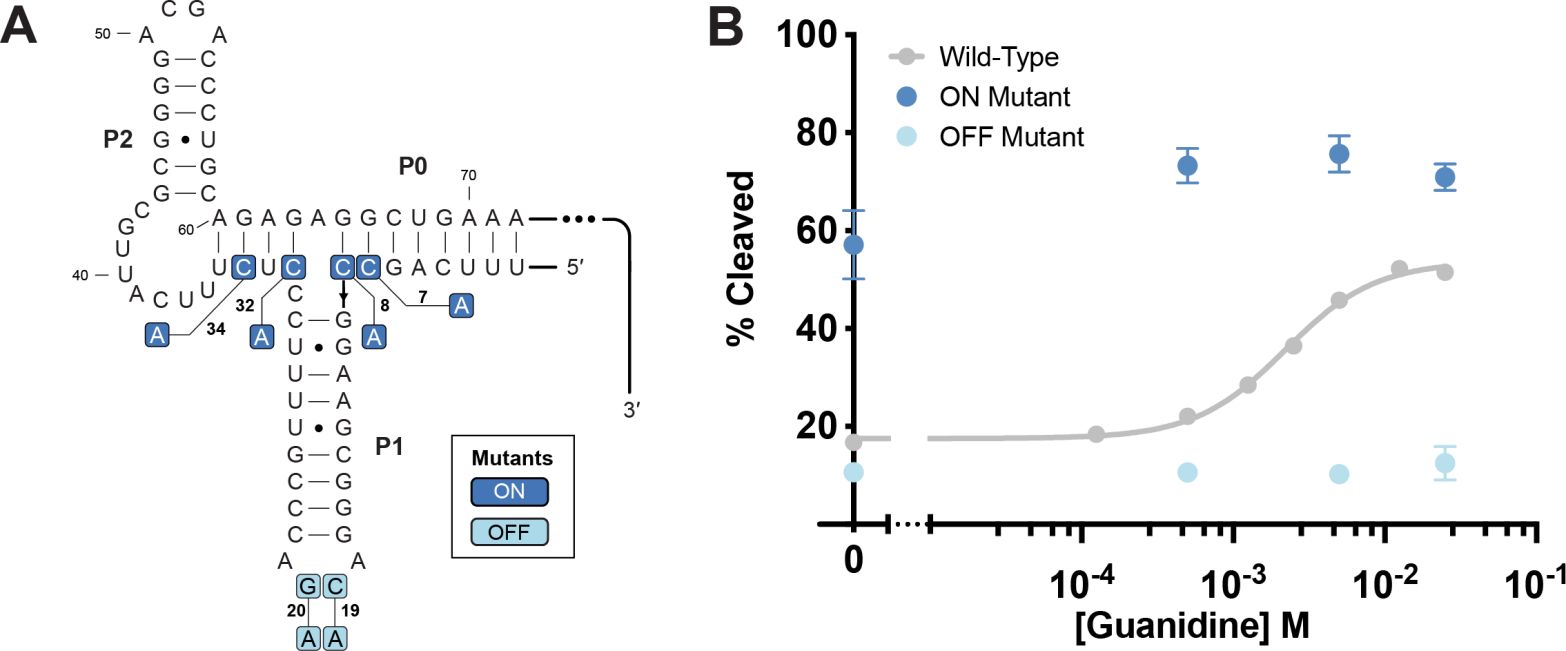
RelE cleavage captures mutational functional perturbations. (**A**) Secondary structure indicating the mutations made for ON and OFF constructs. The anti Shine-Dalgarno nucleotides mutated in the ON mutant are shown in dark blue. The binding site nucleotides mutated in the OFF mutant are shown in light blue. (**B**) The ligand responsiveness of the ON and OFF mutants as compared to the WT sequence.

The RelE assay is also sensitive to sequence-dependent changes in ribosome loading. Mutations were made to the *P. aeruginosa* riboswitch in order to ‘break’ the switch by promoting the formation of either the ON or OFF conformation regardless of ligand concentration. To break the riboswitch ON, mutations were made to the putative P0 helix to inhibit sequestration of the Shine-Dalgarno sequence (Figure 3A). Nucleotides C7, C8, C32 and C34 were mutated to adenines. When subjected to ribosome initiation and RelE cleavage, the ON construct showed no ligand-dependent modulation (Figure 3B, Supplemental Figure S3). Instead, near maximum percent cleaved was maintained across all concentrations. To break the riboswitch OFF, mutations were made to the guanidine binding pocket in L1 to abolish ligand binding (Figure 3A). Nucleotides C19 and G20, which directly contact the ligand and zipper the two binding sites together through a kissing loop interaction (Figure 2B, C), were mutated to adenines. This mutant similarly lost ligand responsiveness. The minimum percent cleaved was observed across all concentrations (Figure 3B, Supplemental Figure S3). Thus, RelE cleavage sensitively reports functional variations in 5′-UTR sequences. These data suggest that RelE cleavage can be used as a read out for ligand-dependent association of a translational riboswitch with bacterial ribosomes.

### RelE cleavage accurately demonstrates switched riboswitch specificity

**Figure 4.**
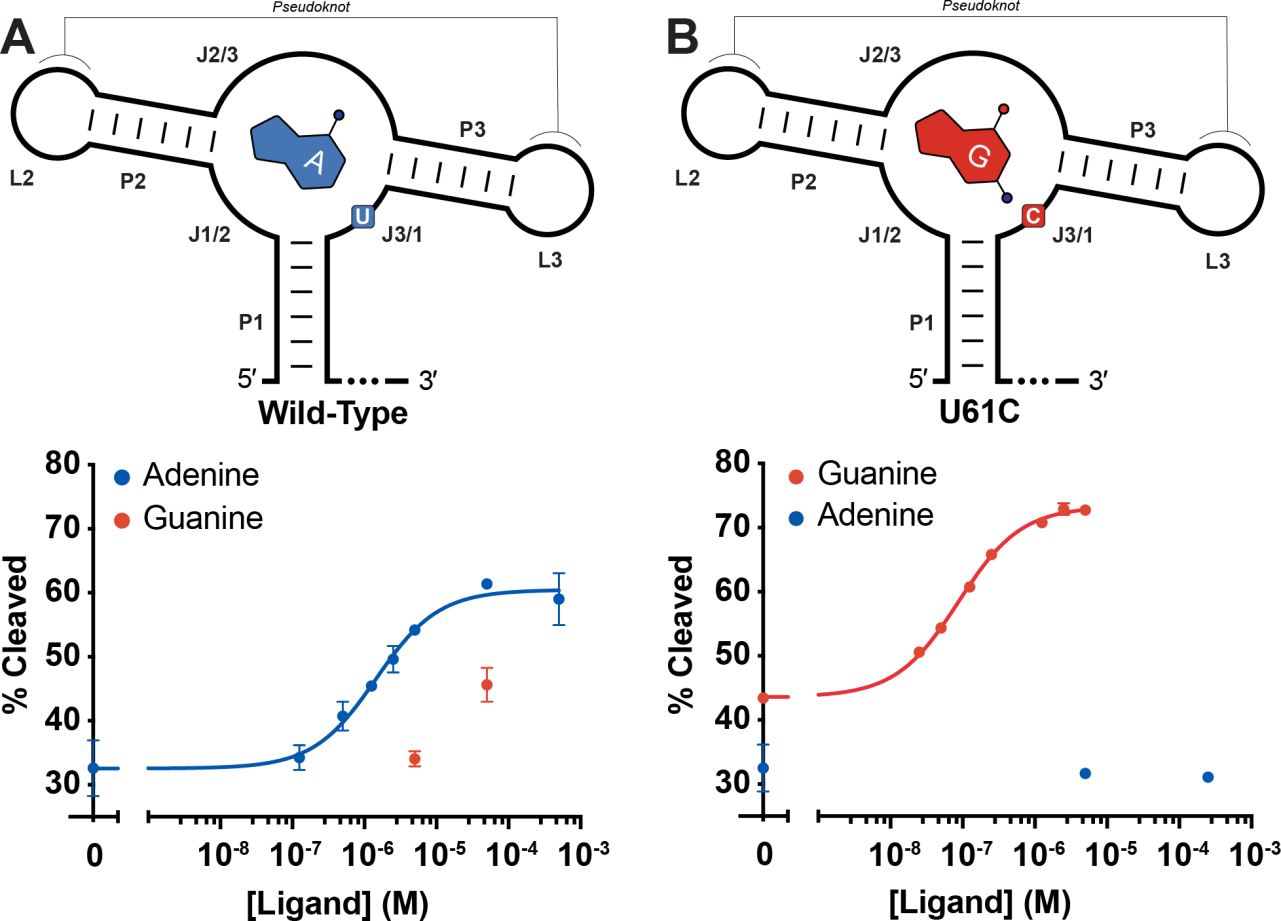
RelE cleavage detects switched riboswitch specificity. (**A**) Secondary structure of the Wild-Type *Vvu* add adenine riboswitch and the RelE cleavage data with both adenine and guanine. Adenine and the specificity nucleotide U61 are shown in blue. (**B**) Secondary structure of the U61C *Vvu* add adenine riboswitch and the RelE cleavage data with both adenine and guanine. Guanine and the mutated specificity nucleotide U61C are shown in red.

We further validated the method with the *Vibrio vulnificus* (*Vvu*) adenine-sensing *add* riboswitch. In the presence of adenine, this translational ON switch promotes translation of the downstream adenine deaminase. It is a member of the well-studied purine riboswitch class (25–27). The core aptamer comprises three helices (P1, P2 and P3) with the ligand binding site situated in the multihelix junction formed by J1/2, J2/3 and J3/1 when loops L2 and L3 form a pseudoknot (25) (Figure 4A). It was originally identified as guanine-binding switch, but a single C to U mutation in J3/1 switches the specificity to adenine (28).

We performed the RelE cleavage assay on the wild-type *Vvu* adenine riboswitch over a range of adenine and guanine concentrations. The percent cleaved at each concentration was fit with Equation (2) (Figure 4A, Supplemental Figure S4). The wild-type *Vvu* sequence responds to adenine with a *K*_1/2_ of 1.4 ± 0.7 μM and an amplitude of 28 ± 2% (*n* = 2). In the presence of guanine, however, the wild-type *Vvu* riboswitch is much less responsive. While a full concentration range could not be performed due to the solubility limit of guanine, the *K*_1/2_ is at least 100-fold weaker (Figure 4A). This is consistent with a previous report that the guanine affinity of the *ydhL* adenine riboswitch is weaker than 10 μM (28). This promiscuity can be attributed to potential wobble base pairing between the guanine ligand and U61.

We then attempted to switch the ligand specificity of the riboswitch from adenine to guanine. We mutated U61 in our wild-type *Vvu* adenine riboswitch to C in order to allow for canonical pairing between the riboswitch and the guanine ligand. The U61C mutant, as expected, lost all measurable response to adenine (Figure 4B). Instead, the U61C *Vvu* riboswitch responded to guanine with a *K*_1/2_ of 87 ± 3 nM and an amplitude of 30 ± 1% (*n* = 2). RelE thus sensitively reports functional differences in riboswitch specificity.

### RelE captures translational differences between eukaryotic 5′-UTR isoforms

**Figure 5.**
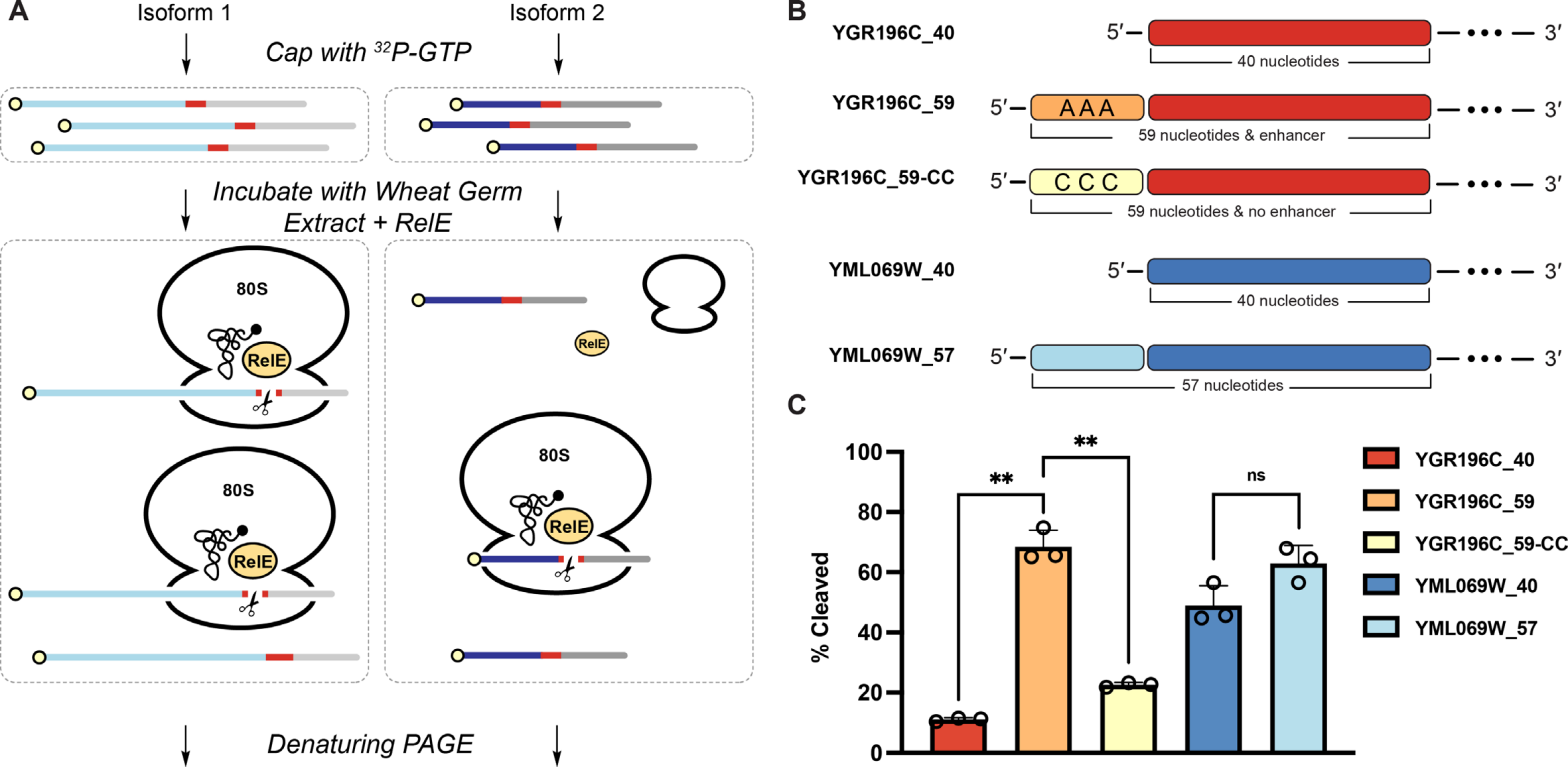
RelE captures differential translation initiation in eukaryotic systems. (**A**) Schematic of RelE Cleavage Assay for Eukaryotic 5′-UTR Isoforms. 5′ ^32^P labeled cap structure is shown in yellow. Site of RelE cleavage is shown in red. Downstream sequence is shown in grey. (**B**) Representation of the various yeast 5′-UTR isoforms tested. (**C**) RelE cleavage of yeast 5′-UTR isoforms. Two-tailed unpaired t-test with Welch's correlation performed within isoform pairs (*n* = 3, ** *P* ≤ 0.01).

We next tested if RelE could be used to monitor the functional differences in translational initiation efficiency between a set of yeast 5′-UTR isoforms. In addition to prokaryotic ribosomes, RelE efficiently cleaves mRNAs loaded onto eukaryotic 80S ribosomes (29). We therefore adapted our previous workflow to suit eukaryotic translational regulators (Figure 5A). A recent high-throughput study has reported functional variations in ribosome initiation between thousands of pairs of yeast 5′-UTR isoforms (8). We selected a pair of isoforms previously identified to be differentially initiated (YGR196C_40 & _59) and a pair of isoforms similar in length, but with no reported differences in translation initiation (YML069W_40 & 57) (Figure 5B). After incubating the radiolabeled isoforms in wheat germ extract supplemented with RelE, we quantified the percent cleaved for each isoform. RelE cleavage requires both the addition of lysate and the addition of RelE (Supplemental Figures S5 and S6). The YGR196C isoform pair shows a significant difference in RelE cleavage (*P* = 0.0024), while the YML069W isoform pair does not (*P* > 0.05) (Figure 5C, Supplemental Figure S7). The long isoform of the YGR196C 5′-UTR (YGR196C_59) has two previously identified (8) putative enhancer sequences (Figure 5B, Supplemental Information) that are potentially responsible for the observed increase in translation initiation. We mutated these A-rich enhancer sequences to poly-C sequences, and observed a significant decrease in RelE cleavage (*P* = 0.0041) (Figure 5C, Supplemental Figure S7A) consistent with reduced translational initiation. Thus, RelE cleavage similarly reports sequence-driven functional differences in translation initiation within a eukaryotic system.

## DISCUSSION

We have developed a RelE cleavage assay for the functional interrogation of translational riboswitches and eukaryotic 5′-UTR isoforms. Since sequence and structural motifs have known ties to translation initiation efficiency, this method provides a quick and straightforward way to analyze the effect of various *cis* RNA regulators, their variants, and reaction conditions on ribosomal loading.

The method cleanly resolves ligand-dependent changes in riboswitch-controlled ribosomal loading through the separation of cleaved and full-length species via gel electrophoresis. We demonstrated this for both an adenine riboswitch and a guanidine riboswitch. Structure-guided mutations to the guanidine riboswitch confirmed the assay's sensitivity to sequence-driven changes in translation initiation. RelE cleavage additionally monitors riboswitch specificity as illustrated by switching an adenine riboswitch's ligand preference to guanine.

This assay fulfills the need for a facile, reliable method to monitor the function of translationally controlled riboswitches. Transcriptionally controlled riboswitches have benefited from the well-established transcription termination assay. Not only has this technique allowed for unambiguous confirmation of transcriptional riboswitches’ regulatory mechanism, but easy integration with sequencing pipelines has facilitated the study of aptamer-expression platform interaction through high-throughput mutational analysis (30). Translationally controlled riboswitches, however, have depended on more cumbersome, less direct techniques. Ribosome toeprinting, for example, has been used to validate ribosome association with the *E. coli thiM* thiamine pyrophosphate (TPP) riboswitch (31). Filter binding has long been used to monitor ribosome-RNA association (32). Ultracentrifugation has been recently employed as a translation initiation assay for yeast 5′-UTRs (8). Fluorescent reporters have also been used to validate riboswitch function (10). RelE provides a snapshot of the ribosome-associated riboswitch population across ligand concentrations while circumventing the need for stringent primer design, laborious washes and centrifugation steps, or fluorescence measurements that are subject to high background levels. This RelE cleavage assay generates quantitative data regarding a riboswitch's sensitivity (*K*_1/2_) as well as its dynamic range (amplitude) via a standard gel-based readout.

Since the method utilizes *in vitro* transcribed and refolded RNA, the assay is most useful for thermodynamically rather than kinetically driven regulatory mechanisms. While kinetic translational riboswitches have been reported (33), many translationally controlled riboswitches are expected to function thermodynamically so that they can continue their regulatory role over the lifetime of the transcript. The *Vvu add* adenine riboswitch has been characterized previously as a thermodynamic switch whose function is separate from transcriptional dynamics (34). We have shown here that the *Pae* Guanidine-II riboswitch functions in a similar manner.

The assay also has the potential to refine the resolution of previous work determining ribosome occupancy on various yeast 5′ leaders. We have demonstrated that RelE sensitively reports isoform-specific differences in translation initiation, but it also identifies the position of the A site to nucleotide resolution. While our constructs include UAG stop codons as RelE cleavage sites, RelE has been shown to cleave a wide variety of sequences. RelE cleavage could thus be used with endogenous or modestly engineered sequences to determine the contribution of upstream AUGs to the ribosome occupancy of relevant transcripts.

Since the effects of variable conditions can be conveniently examined, this method may also be expanded to other known *cis*-acting 5′-UTR motifs. Variant riboswitches can be screened against various ligands, RNA thermometers can be screened across various temperatures, and enhancer and repressor elements can be screened against various RNA binding proteins.

Beyond condition space, sequence space can also be easily explored. Given its quantitative nature and seamless integration into existing high-throughput pipelines, RelE cleavage can be readily applied to complex libraries of 5′ leader sequences. With a sequencing-based readout, this method may be expanded to libraries of endogenous 5′ leader isoforms to provide broad insight into translational initiation across the transcriptome. Starting with a mutant library, however, RelE cleavage may also provide fine-grained detail of regulatory motifs such as viral internal ribosome entry sites (IRESes) through mutational analysis. RelE cleavage can thus reveal a plethora of information about translation initiation in different domains of life.

## DATA AVAILABILITY

Representative gels are included in the text and supplemental. All gel images are available from the corresponding author upon request.

## Supplementary Material

gkac614_Supplemental_File
