# Supplementary material for: Efficient quantitative monitoring of translational initiation by RelE cleavage
Source: Nucleic Acids Res. 2022 Jul 25;50(18):e105. doi: 10.1093/nar/gkac614 (PMC9561414; doi:10.1093/nar/gkac614)
Supplement: gkac614_Supplemental_File [file gkac614_supplemental_file.pdf]

Efficient Quantitative Monitoring of Translational Initiation by RelE Cleavage

Caroline M. Focht and Scott A. Strobel

Supplementary Information

Supplementary Table 1. RNA sequences used.
*Mutations to wild-type sequences shown in red. Stop codon for RelE cleavage underlined.
**Enhancer elements bold and underlined

Name	Sequence
Pae Gdm WT	UUUCAGCCGGAAGCGGGACGACCCGUUUUCCCUCUUUCAUUGCGCGGGGACG<br>ACCCUGCAGAGAGGCUGAAAAUGUAGUGGAUCUAUCUCUUGCUCGUA
Pae Gdm ON *	UUUCAGAAGGAAGCGGGACGACCCGUUUUCCAUAUUUCAUUGCGCGGGGACG<br>ACCCUGCAGAGAGGCUGAAAAUGUAGUGGAUCUAUCUCUUGCUCGUA
Pae Gdm OFF *	UUUCAGCCGGAAGCGGGAAAACCCGUUUUCCCUCUUUCAUUGCGCGGGGACG<br>ACCCUGCAGAGAGGCUGAAAAUGUAGUGGAUCUAUCUCUUGCUCGUA
Vvu add	GCUUCAUAUAAUCCUAAUGAUAUGGUUUGGGAGUUUCUACCAAGAGCCUUAA<br>ACUCUUGAUUAUGAAGUCUGUCGCUUUAUCCGAAAUUUUAUAAAGAGAAGAC<br>UCAUGUAGAAUUACUUUGACCUGCCG
Vvu add U61C *	GCUUCAUAUAAUCCUAAUGAUAUGGUUUGGGAGUUUCUACCAAGAGCCUUAA<br>ACUCUUGACUAUGAAGUCUGUCGCUUUAUCCGAAAUUUUAUAAAGAGAAGAC<br>UCAUGUAGAAUUACUUUGACCUGCCG
YGR196C Short Isoform	GGUUUGAUCAUUACCCUUUUUCUGGAAAGCGGAUAUUUAUUAUGUAGACUG<br>AACAGGUCGGUAGAAAGAAA
YGR196C Long Isoform **	GAUU**AU**AGUU**AAAA**GGGACUGUUUGAUCAUUACCCUUUUUCUGGAAAGCGG<br>AUAUUUAUUAUGUAGACUGAACAGGUCGGUAGAAAGAAA
YGR196C Long Isoform No Enhancer*	GAUUCCAGUUCCCCGGGACUGUUUGAUCAUUACCCUUUUUCUGGAAAGCGGA<br>UAUUUAUUAUGUAGACUGAACAGGUCGGUAGAAAGAAA
YML096W Short Isoform	GACAAGUGAAGGAACAAUCUAGUAUUGUUGAACAAGAAUUAAUGUAGACCGA<br>CUUUGAUAGAAUUUACUUGAA
YML096W Long Isoform	GAGAAGGAAAUACCGUUAACAAGUGAAGGAACAAUCUAGUAUUGUUGAACAA<br>GAAUUAAUGUAGACCGACUUUGAUAGAAUUUACUUGAA

Supplementary Table 2. Oligos used.

Name	Sequence
Vvu_add	CGGCAGGTCAAAGTAATTCTACATGAGTCTTCTCTTTATAAAATTTCGGATAAA GCGACAGACTTCATAATCAAGAGTTTAAGGCTCTTGGTAGAAACTCCCAAACCA TATCATTAGGATTATATGAAGCTATAGTGAGTCGTATTA
Vvu_add_U61C	CGGCAGGTCAAAGTAATTCTACATGAGTCTTCTCTTTATAAAATTTCGGATAAA GCGACAGACTTCATAGTCAAGAGTTTAAGGCTCTTGGTAGAAACTCCCAAACCA TATCATTAGGATTATATGAAGCTATAGTGAGTCGTATTA
Pae_Gdm_WT	TACGAGCAAGAGATAGATCCACTACATTTTCAGCCTCTCTGCAGGGTCGTCCCC GCGCAATGAAAGAGGGAAAACGGGTCGTCCCGCTTCCGGCTGAAACCTATAGT GAGTCGTATTA
Pae_Gdm_ON	TACGAGCAAGAGATAGATCCACTACATTTTCAGCCTCTCTGCAGGGTCGTCCCC GCGCAATGAAATATGGAAAACGGGTCGTCCCGCTTCCTTCTGAAACCTATAGTG AGTCGTATTA
Pae_Gdm_OFF	TACGAGCAAGAGATAGATCCACTACATTTTCAGCCTCTCTGCAGGGTCGTCCCC GCGCAATGAAAGAGGGAAAACGGGTTTTCCCGCTTCCGGCTGAAACCTATAGT GAGTCGTATTA
T7 Promoter Duplex	TAATACGACTCACTATAG
YGR196C_40	TTTCTTTCTACCGACCTGTTCAGTCTACATAATAAATATCCGCTTTCCAGAAAAA GGGTAATGATCAAACCTATAGTGAGTCGTATTA
YGR196C_59	TTTCTTTCTACCGACCTGTTCAGTCTACATAATAAATATCCGCTTTCCAGAAAAA GGGTAATGATCAAACAGTCCCTTTTAACTATAATCTATAGTGAGTCGTATTA
YGR196C_59_CC	TTTCTTTCTACCGACCTGTTCAGTCTACATAATAAATATCCGCTTTCCAGAAAAA GGGTAATGATCAAACAGTCCCGGGGAACTGGAATCTATAGTGAGTCGTATTA
YML069W_40	TTCAAGTAAATTCTATCAAAGTCGGTCTACATTAATTCTTGTTCAACAATACTAG ATTGTTCCTTCACTTGTCTATAGTGAGTCGTATTA
YML069W_57	TTCAAGTAAATTCTATCAAAGTCGGTCTACATTAATTCTTGTTCAACAATACTAG ATTGTTCCTTCACTTGTTAACGGTATTTCCTTCTCTATAGTGAGTCGTATTA

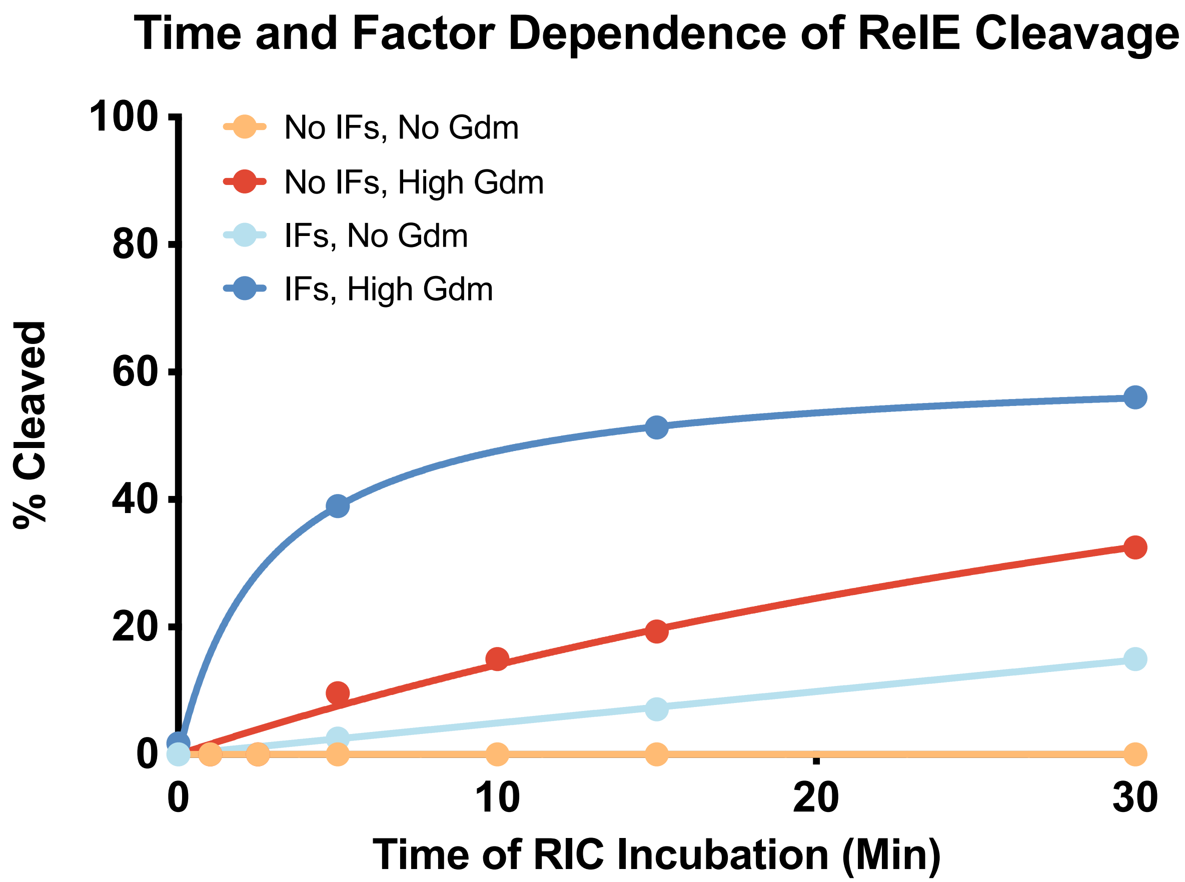

Supplementary Figure 1. Time course of RelE cleavage with and without purified initiation factors at low and high guanidine concentrations. Initiation factors clearly increase rate of ribosome association as read out by RelE cleavage. The data were fit with the following hyperbola: $Y = Y_{Max} [X / (K + X)]$

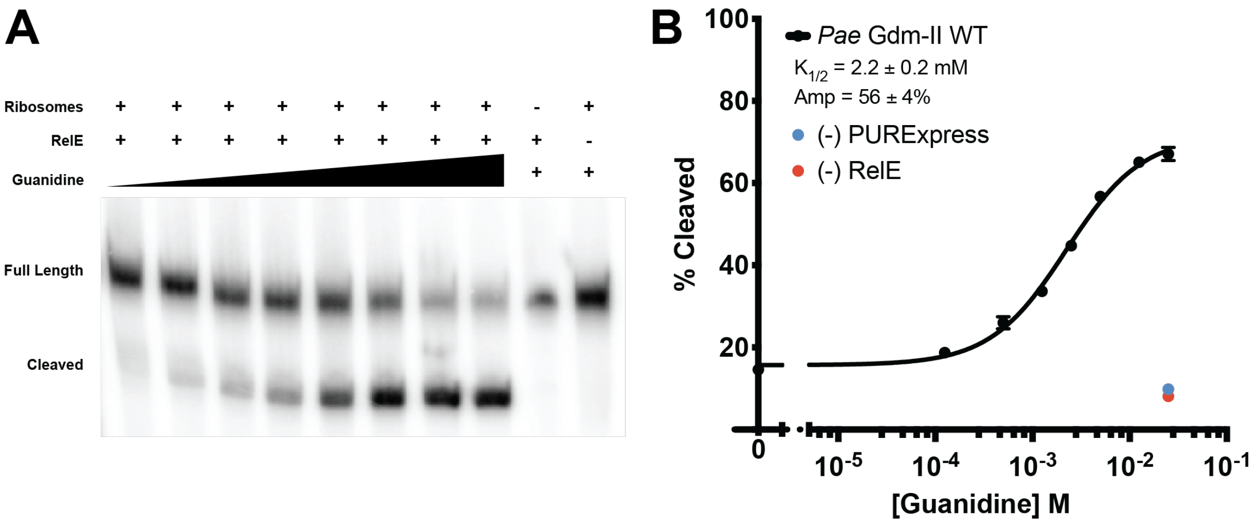

Supplementary Figure 2. RelE cleavage in PURExpress ΔRF123. A) Representative gel of RelE cleavage of the Pae *Gdm-II riboswitch across guanidine concentrations (0 – 25 mM) and with no ribosome and no RelE controls. B) Response profile of the* Pae *Gdm-II riboswitch in PURExpress ΔRF123 (n = 2). The data were fit with the Hill equation: $Y = Amp\ [\ X^n / (K^n + X^n)] + Y_{min}$.*

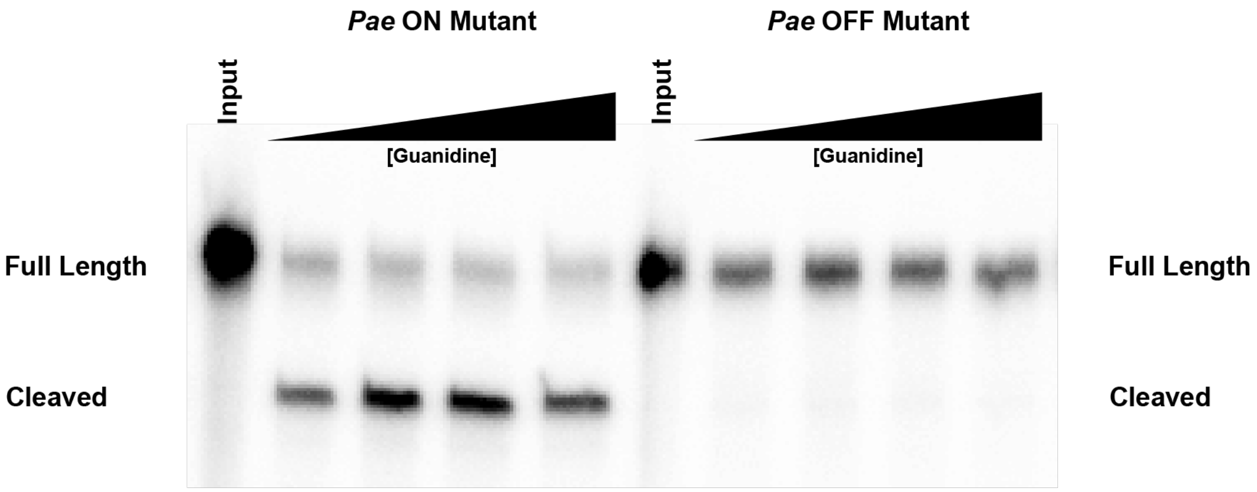

Supplementary Figure 3. Representative gel separation of full-length and cleaved species for the Pae *ON and OFF mutants over four concentrations of guanidine (0 – 25 mM). Initiation done with purified components.*

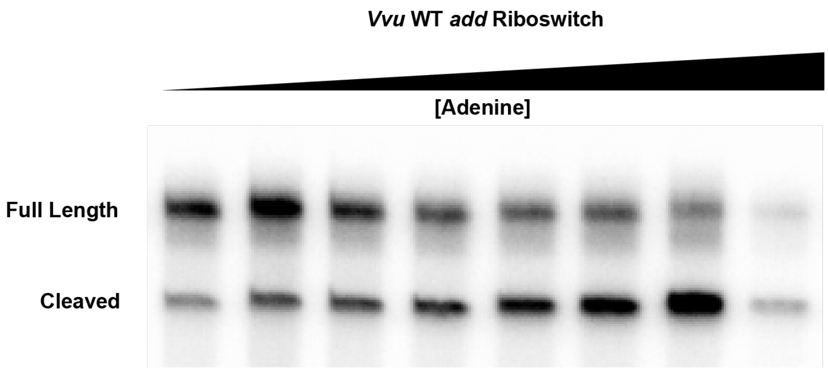

Supplementary Figure 4. Representative gel separation of full length and cleaved species for the Vvu *WT* add *riboswitch over eight concentrations of adenine (0 – 500 μM). Initiation done with purified components.*

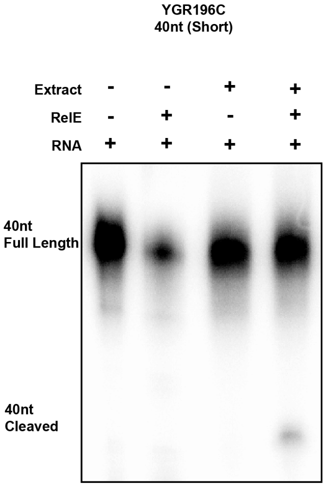

Supplementary Figure 5. Gel separation of full length and cleaved species for the YGR169C_40 construct alongside wheat germ extract (Promega) and RelE controls.

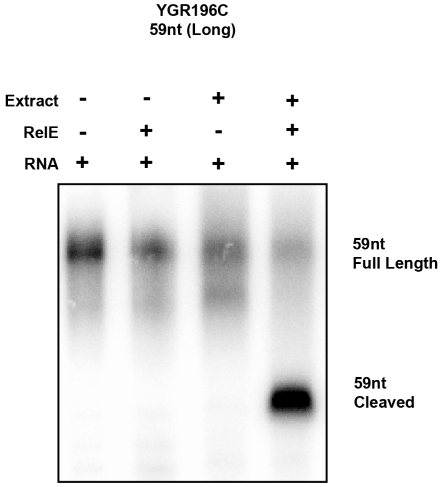

Supplementary Figure 6. Gel separation of full length and cleaved species for the YGR169C_59 construct alongside wheat germ extract (Promega) and RelE controls.

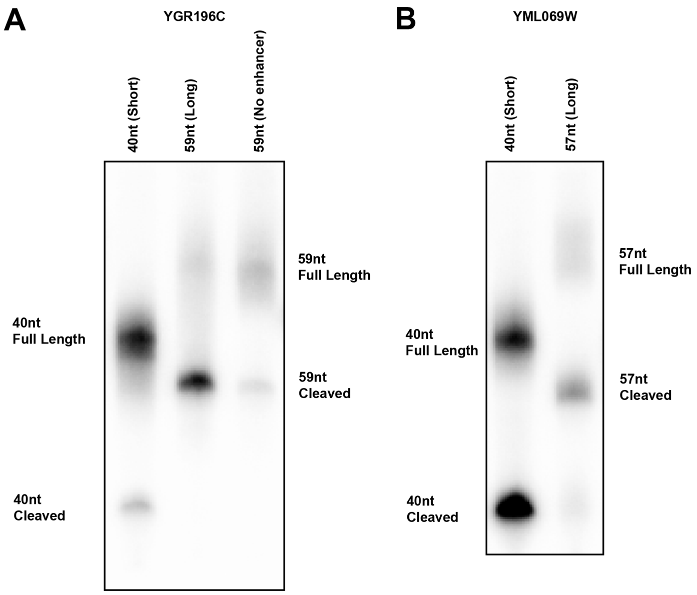

Supplementary Figure 7. Representative gels of yeast 5' UTR isoform RelE cleavage in wheat germ extract (Promega). A) Cleavage of YGR196C_40, YGR196C_59, and YGR196C_59_CC B) Cleavage of YML069W_40 and YML069W_57
